# Biomimetic bone-vessel interface-on-a-chip for simulating periodontal physiological and pathological microenvironment

**DOI:** 10.1093/rb/rbaf111

**Published:** 2025-10-28

**Authors:** Chen Chen, Jianan Hui, Tian Tian, Laidi Jin, Xue Li, Bingcheng Lin, Guowu Ma, Hongju Mao, Huiying Liu

**Affiliations:** School of Stomatology, Dalian Medical University, Dalian 116044, China; Academician Laboratory of Immune and Oral Development & Regeneration, Dalian Medical University, Dalian 116044, China; Dalian Medical University School and Hospital of Stomatology, Dalian 116000, China; State Key Laboratory of Transducer Technology, Shanghai Institute of Microsystem and Information Technology, Chinese Academy of Sciences, Shanghai 200050, China; State Key Laboratory of Transducer Technology, Shanghai Institute of Microsystem and Information Technology, Chinese Academy of Sciences, Shanghai 200050, China; State Key Laboratory of Transducer Technology, Shanghai Institute of Microsystem and Information Technology, Chinese Academy of Sciences, Shanghai 200050, China; School of Stomatology, Dalian Medical University, Dalian 116044, China; Academician Laboratory of Immune and Oral Development & Regeneration, Dalian Medical University, Dalian 116044, China; Dalian Medical University School and Hospital of Stomatology, Dalian 116000, China; State Key Laboratory of Transducer Technology, Shanghai Institute of Microsystem and Information Technology, Chinese Academy of Sciences, Shanghai 200050, China; School of Stomatology, Dalian Medical University, Dalian 116044, China; Academician Laboratory of Immune and Oral Development & Regeneration, Dalian Medical University, Dalian 116044, China; Dalian Medical University School and Hospital of Stomatology, Dalian 116000, China; State Key Laboratory of Transducer Technology, Shanghai Institute of Microsystem and Information Technology, Chinese Academy of Sciences, Shanghai 200050, China; Department of Biotechnology, Dalian Institute of Chemical Physics, Chinese Academy of Sciences, Dalian 116023, China; School of Stomatology, Dalian Medical University, Dalian 116044, China; Academician Laboratory of Immune and Oral Development & Regeneration, Dalian Medical University, Dalian 116044, China; Dalian Medical University School and Hospital of Stomatology, Dalian 116000, China; State Key Laboratory of Transducer Technology, Shanghai Institute of Microsystem and Information Technology, Chinese Academy of Sciences, Shanghai 200050, China; School of Stomatology, Dalian Medical University, Dalian 116044, China; Academician Laboratory of Immune and Oral Development & Regeneration, Dalian Medical University, Dalian 116044, China; Dalian Medical University School and Hospital of Stomatology, Dalian 116000, China

**Keywords:** organ-on-a-chip, microfluidic technology, bone-vessel interface, inflammation

## Abstract

Periodontitis is a chronic inflammatory disease affecting periodontal supporting tissues. Untreated, it causes irreversible alveolar bone destruction, ultimately leading to tooth loss. Periodontitis-associated pathogenic bacteria/metabolites and pro-inflammatory factors can initiate or exacerbate systemic disease through the circulation. Endothelial cells, forming the interface between circulation and tissues, play a key role in disease progression. As microfluidic organ chips enable the establishment of tissue–tissue interfaces and simulation of the *in vivo* microenvironment, we constructed a bone-vessel interface-on-a-chip. Within this physiological model, human bone marrow mesenchymal stem cells (hBMSCs) and human umbilical vein endothelial cells (HUVECs) were successfully co-cultured with high viability. HUVECs formed a confluent monolayer exhibiting selective vascular permeability. Osteo-differentiated hBMSCs expressed alkaline phosphatase, secreted bone-related proteins, and formed mineralized deposits. By introducing the *Porphyromonas gingivalis* (*P. gingivalis*) metabolite LPS and the pro-inflammatory factor TNF-α, we established an inflammatory microenvironment. The chip model subsequently exhibited vascular endothelial intercellular junction disruption, upregulated adhesion protein expression, enhanced monocyte adhesion, impaired vascular endothelial barrier function, and reduced bone-related protein expression. These results demonstrate that bone-vessel interface-on-a-chip can effectively study the effects of periodontitis metabolites and pro-inflammatory factors on the vascular barrier and bone tissue through controlled integration of biochemical and biophysical cues. This model provides a robust platform for investigating endothelial cell-targeted therapies for inflammatory diseases, including periodontitis and associated systemic diseases.

## Graphical Abstract

**Figure:**
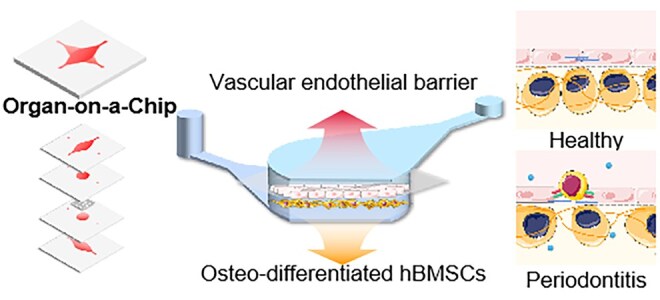

## Introduction

**Figure 1.**
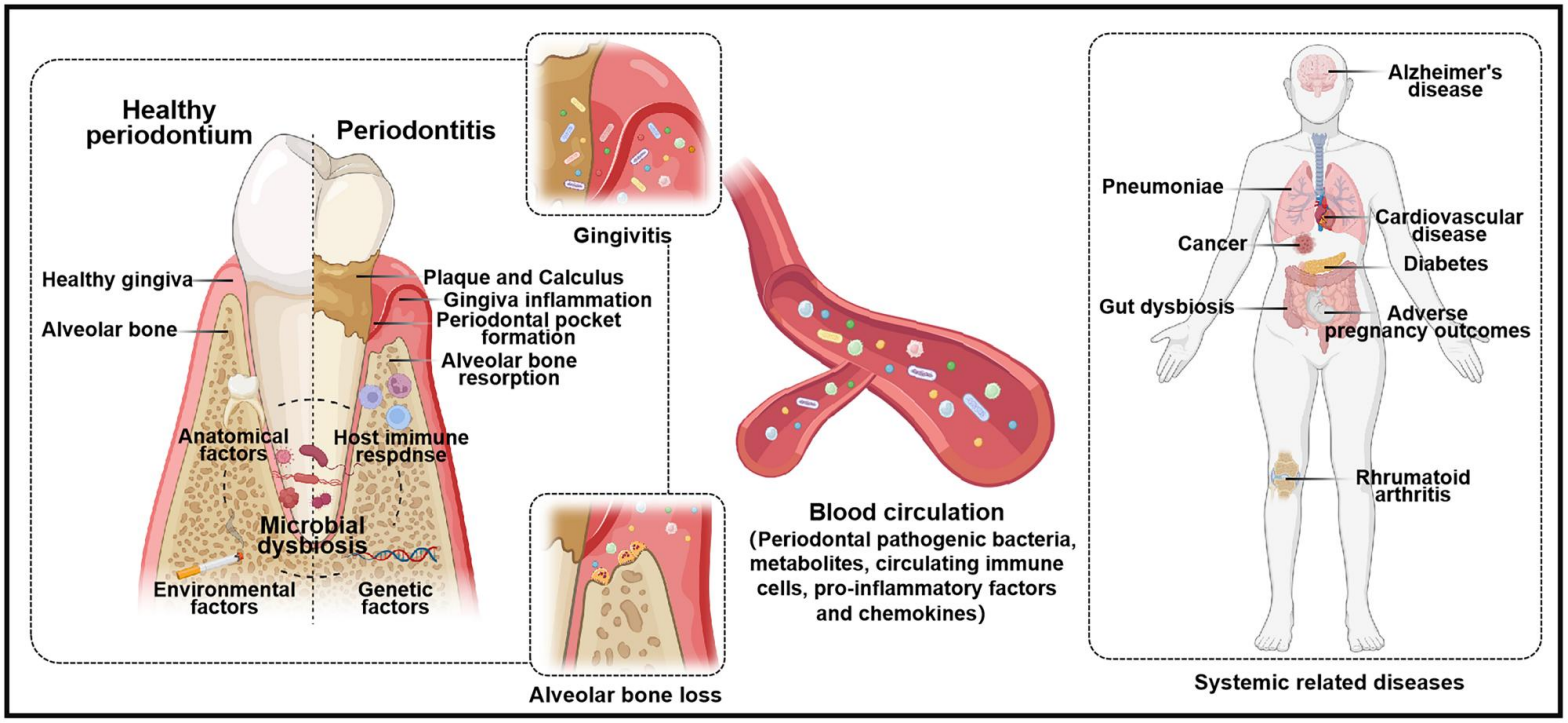
Association between periodontal disease and systemic diseases. Figure created with BioRender.com.

Periodontitis is a chronic inflammatory disease driven by microbial pathogens, with local anatomical features, host immune response, and genetic factors serving as contributing determinants [1]. Gingivitis, typically preceding periodontitis, represents a reversible inflammatory reaction that is self-limiting upon plaque removal. If untreated, it progresses to an advanced stage characterized by irreversible alveolar bone destruction, leading to tooth loosening, displacement, and eventual loss [2]. Consequently, periodontitis significantly impairs chewing function, aesthetics, and quality of life. Beyond local effects, periodontitis can induce or exacerbate systemic diseases through dual mechanisms: direct dissemination of pathogenic bacteria/metabolites, and indirect induction of pro-inflammatory cascades via the circulatory system. Established associations link periodontitis to cardiovascular disease, diabetes, osteoporosis, and Alzheimer’s disease through these pathways (Figure 1) [3–5]. Periodontal pathogens such as *Porphyromonas gingivalis* (*P. gingivalis*) persistently colonize dental plaque, secreting virulence factors including lipopolysaccharide (LPS) and collagenase. These metabolites disrupt host immune homeostasis, triggering excessive production of pro-inflammatory mediators, such as tumor necrosis factor-α (TNF-α), interleukins (ILs), prostaglandin E_2_ (PGE_2_), and matrix metalloproteinases (MMPs) [6, 7]. Under the combined influence of microbial and host-derived inflammatory mediators, gingival inflammation—initially confined to the epithelium and connective tissue—spreads systemically via blood circulation. This drives advanced alveolar bone resorption and functional damage to distant organs. Blood vessels serve as critical interfaces between circulation and peripheral tissues, characterized by structures regulating vital physiological processes [8]. Endothelial cells form a barrier that acts as the gatekeeper of the tissue microenvironment to maintain homeostasis [9]. As the first line of defense, they directly participate in responses to inflammation and infection. Endothelial activation or dysfunction is intricately associated with chronic inflammatory diseases, playing a pivotal role in disease onset and progression [8]. Therefore, endothelial cells represent promising targets for anti-inflammatory therapies and systemic interventions. While physiologically essential for tissue healing, persistent release of inflammatory mediators inevitably induces tissue damage [10]. LPS, a major component of the outer membrane of Gram-negative bacteria like *P. gingivalis*, exerts potent pathogenic effects in periodontal tissues [11]. Upon entering circulation, it triggers septic responses, potently activating leukocytes and promoting inflammatory mediator release [12]. TNF-α, a key pro-inflammatory cytokine, is significantly upregulated in periodontal tissues, serum, and gingival crevicular fluid of periodontitis patients, correlating with periodontitis activity [13, 14]. Elevated circulating levels of LPS and TNF-α drive adhesion molecule and chemokine expression, leading to vascular barrier dysfunction and inflammatory leukocyte transendothelial migration (TEM), thereby damaging downstream organs [15–17]. Therefore, establishing an *in vitro* model that recapitulates the anatomical structure and physiological function of periodontal tissue while simulating blood circulation is crucial for exploring periodontitis pathogenesis and developing strategies to prevent periodontitis, related systemic diseases, and ultimately maintain periodontal and systemic health.

Current *in vitro* models for periodontitis research primarily include animal models, 2D cultures, and 3D cultures. While animal models remain the gold standard for preclinical drug screening and proof-of-concept therapeutic studies due to their high reproducibility, ethical concerns persist [18]. Furthermore, inherent species differences in physiology and metabolism limit their translational value. These models also often lack sufficient temporal resolution and sensitivity, hindering detailed mechanistic investigation of cellular interactions and physiological processes [19, 20]. Compared to simple 2D monolayer cultures, advanced 3D models—such as organotypic cultures, spheroids, and scaffold-based systems—better recapitulate the *in vivo* microenvironment for studying cell–cell and cell–extracellular matrix (ECM) interactions [21]. Nevertheless, 3D models present challenges in sampling from distinct tissue compartments. Critically, the material and structure constraints of traditional porous supports in the 3D models impede the implementation of perfusion culture, mechanical force simulation, and multi-organ interaction studies *in vitro* [22].

Organ-on-a-chip (OoC) technology utilizes microfluidic cell culture devices fabricated with microfabrication techniques. These devices incorporate perfusable microchambers that recapitulate tissue/organ-level physiology by culturing living cells [19, 23]. OoC platforms can reproduce multicellular structure, tissue–tissue interfaces, physicochemical microenvironment, and vascular perfusion, enabling intercellular communication, and the construction of integrated functional tissue/organ units *in vitro*. Unlike traditional models, OoC platforms permit real-time, high-resolution microscopic imaging and biochemical/metabolic analysis of living cells within a functional human tissue/organ context [22, 23]. By enabling precise control over single or combined cellular, molecular, chemical, and biophysical parameters, OoC platforms empower researchers to dissect their contributions to disease pathogenesis, progression, and therapeutic responses [24, 25]. For periodontal research, OoC systems offer the potential to model the complex periodontal microenvironment, encompassing structural/cellular complexity, biochemical/mechanical crosstalk, and microbial–host interactions [26–29]. These systems facilitate *in vitro* reconstruction of oral mucosa [30, 31], gingiva [32, 33], and periodontal ligament [28, 34] by integrating multiple cell types within structured ECM. Specific chip architectures address current challenges in simulating oral physiological/pathological processes: basic single-chamber structures model the effect of salivary mechanical forces on plaque biofilms [35]; multi-array structures enable multifactorial high-throughput screening (e.g. strain combinations [36], sucrose concentrations [37]); parallel-chamber structures (incorporating horizontal micropillar arrays and vertical porous membranes) simulate native tissue scaffolding to investigate pathophysiological processes [38, 39]; serial-chamber structures simulate physiological processes of immunological and digestive systems [40, 41]. However, few studies have specifically investigated the effects of periodontal pathogen metabolites and pro-inflammatory factors on both periodontal bone tissue and vascular endothelial barrier. Current bone-related microfluidic chips primarily include vascularized bone tissue chips, bone marrow chips, cancer bone metastasis chips, and bone cell mechanical mechanotransduction chips [42–45]. These studies demonstrate that OoC technology can serve as an *in vitro* model of the periodontal microenvironment, enabling evaluation of the periodontal bone tissue and vascular endothelial barrier. Importantly, OoC systems address key limitations of current preclinical models and static *in vitro* platforms in simulating cellular and tissue interactions under human physiological and pathological conditions. Collectively, these findings highlight the potential of OoC systems to model periodontal bone-vessel crosstalk and recapitulate the periodontitis microenvironment.

Here, we designed and constructed a microfluidic chip device simulating the bone-vessel interface, incorporating key histological structures and major physiological functions of bone. Human bone marrow mesenchymal stem cells (hBMSCs) and human umbilical vein endothelial cells (HUVECs) were co-cultured within this platform to establish a functional bone-vessel interface. We evaluated vascular endothelial barrier morphology and permeability, bone-related protein expression, and osteoblast tissue mineralization. Furthermore, leveraging this physiological model, we simulated periodontitis-induced effects on the vascular barrier and downstream bone tissue by applying *P. gingivalis* metabolite LPS (direct action) and pro-inflammatory cytokine TNF-α (indirect action via circulation). This chip provides a novel platform for investigating mechanistic insights and therapeutic strategies for periodontitis and its associated systemic diseases.

## Materials and methods

### Device assembly and operation

**Figure 2.**
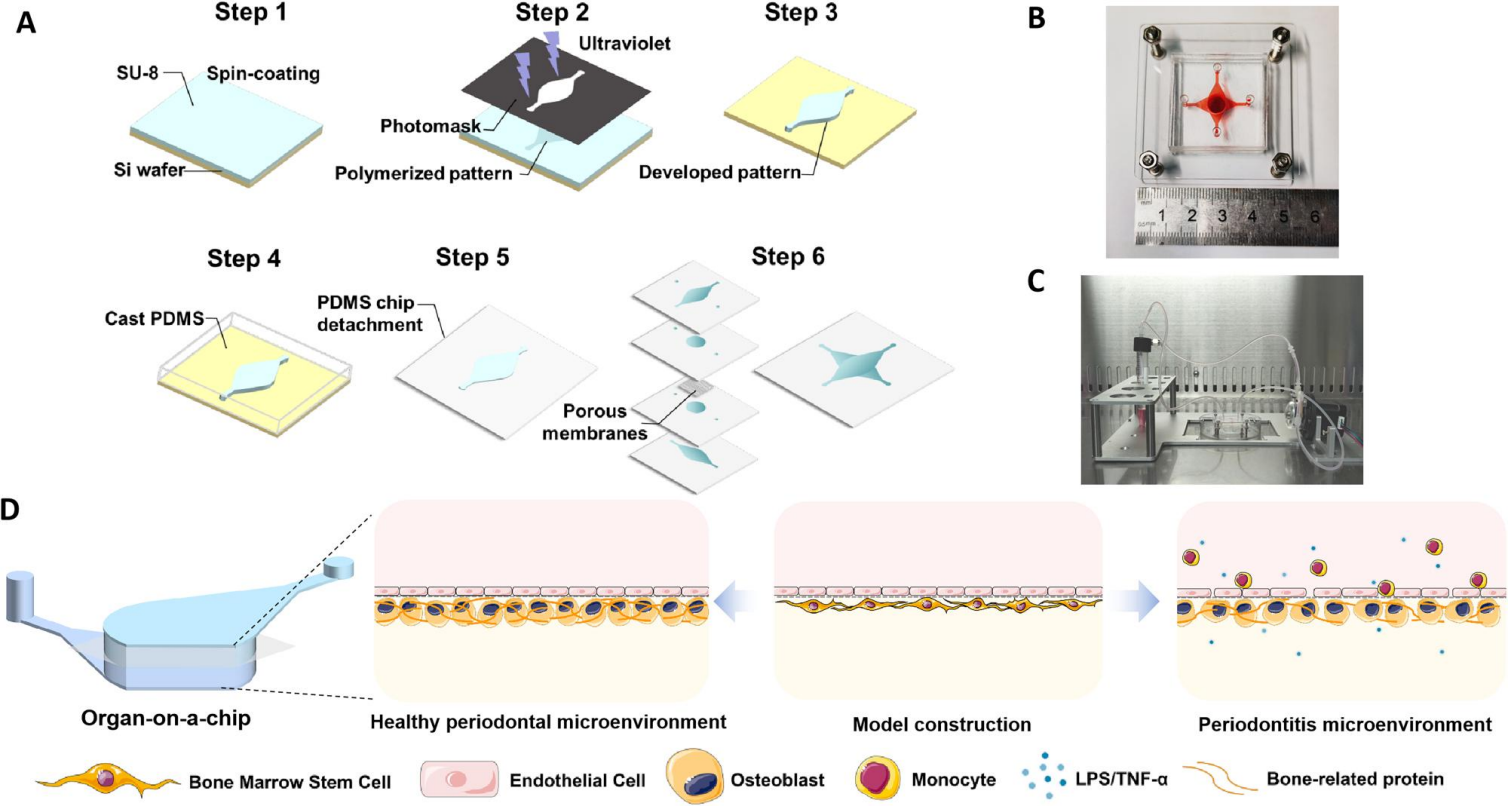
Biologically inspired design and construction of a bone-vessel interface-on-a-chip. (**A**) Microfluidic chip manufacturing process. (**B**) Image of microfluidic chip viewed from above. (**C**) Chip perfusion connection physical image. (**D**) Completion of periodontal physiological and pathological microenvironment by bone-vessel interface-on-a-chip.

The microfluidic device was fabricated using SU8-3050 negative photoresist (Kayaku Advanced Materials, Westborough, MA, USA) and polydimethylsiloxane (PDMS, Dow Corning, Midland, MI, USA) via standard soft lithography and microfabrication techniques (Figure 2A). The device comprised upper and lower chambers separated by a porous membrane (Sterlitech, Auburn, WA, USA), each featuring independent inlet/outlet ports and interconnecting channels. The central cell culture chamber was 6 mm in diameter, with the channel 1 mm wide and liquid inlet/outlet ports 1.5 mm in diameter. A triangular buffer structure was integrated at the chamber-channel junctions. Following 40 s plasma treatment, the porous membrane and PDMS layers were irreversibly bonded. The final assembly consisted of four PDMS layers and the porous membrane (Figure 2B). To ensure stable sealing, the device was secured between two polymethyl methacrylate plates using screws. The assembled chip was connected to a peristaltic pump and medium reservoir for operation (Figure 2C).

### Design and fabrication of bone-vessel interface-on-a-chip

The hBMSCs (Cyagen, Suzhou, Jiangsu, China) were cultured in α-MEM medium (Hyclone, Logan, UT, USA) supplemented with 10% fetal bovine serum (FBS, Gibco, Grand Island, NY, USA) and 1% Penicillin-Streptomycin (P/S, Hyclone, Logan, UT, USA). HUVECs (ATCC, Manassas, VA, USA) were cultured in ECM medium (ScienCell, San Diego, CA, USA) containing 10% FBS and 1% P/S. When confluence reached >90%, cells were digested with 0.25% trypsin-ethylene diamine tetraacetic acid (Trypsin-EDTA, Gibco, Grand Island, NY, USA) at 37°C for 1 min and subcultured.

Bone-vessel interface physiological chip was then constructed. Firstly, fresh hBMSC medium was introduced into the lower chamber to remove excess fibronectin (10 μg/mL; Sigma-Aldrich, St Louis, MO, USA), and hBMSC suspension (5 × 10^4^ cells/mL) was added. Then the chip was inverted to facilitate cell adhesion to the porous membrane underside. When cells reached >90% confluence (3 days), medium was replaced with osteogenic differentiation medium containing 10 mM β-Glycerophosphate, 50 mg/L ascorbic acid, and 0.1 μM dexamethasone (Coolaber, Beijing, China). Subsequently, fibronectin coating (10 μg/mL; Sigma-Aldrich, St Louis, MO, USA) in the upper chamber was replaced with endothelial cell medium. HUVECs (1 × 10^5^ cells/mL) were added to the upper chamber and cultured until a confluent monolayer barrier structure was formed (2–3 days). To establish an inflammatory microenvironment, LPS (from *P. gingivalis*, ultrapure; InvivoGen, San Diego, CA, USA) or TNF-α (Sigma-Aldrich, St Louis, MO, USA) was applied. Chip medium was replaced with complete medium containing 10 μg/mL LPS or 20 ng/mL TNF-α, and samples were evaluated at 1, 7, or 14 days post-stimulation. All cell culture and stimulation procedures were performed at 37°C under 5% CO_2_.

The perfusion velocity was determined by calculating fluid shear stress (τ) using the formula:


$$\tau =\frac{6\mu Q}{h^{2}w}$$

where μ is medium viscosity, *Q* is flow rate, *w* is channel width, *h* is channel height. To ensure physiological shear stress at the narrowest channel region, 5 μL/min was selected as the endothelial-side perfusion rate.

### Cell live/dead assay

To analyze cell status during culture and evaluate chip feasibility, the Live/Dead Cell Staining Kit (Invitrogen, Carlsbad, CA, USA) was used to assess viability. Calcein AM can pass through the cell membrane of living cells to produce green fluorescence. EthD-1 can enter dead cells and combine with nucleic acids in cells to produce red fluorescence. A staining solution was prepared by adding 0.5 µL of Calcein AM and 2 µL of EthD-1 to 1 mL of serum-free cell basal medium. After washing cell-loaded chip with PBS, the solution was applied to upper-layer endothelial cells and lower-layer mesenchymal stem cells. After incubating at 37°C for 10 min in the dark, images were acquired using a Nikon fluorescence microscope.

### Immunofluorescent staining

To characterize protein expression in endothelial cells and mesenchymal stem cells within the bone-vessel interface-on-a-chip, immunofluorescence staining was performed for tissue-specific markers. Both cell types were fixed with 4% formaldehyde for 10 min, permeabilized with 0.1% Triton X-100 for 10 min at room temperature (RT), and blocked with 3% bovine serum albumin for ≥30 min at RT. Following blocking, cells were incubated with primary antibodies. VE-cadherin (Invitrogen, Carlsbad, CA, USA) was labeled with mouse monoclonal antibody at 1:100 dilution. Collagen type I (COL-I) (Abcam, Cambridge, UK) was labeled with rabbit monoclonal antibody at 1:250 dilution. OCN (Invitrogen, Carlsbad, CA, USA) was labeled with rabbit polyclonal antibody at 1:100 dilution. OPN (Invitrogen, Carlsbad, CA, USA) was labeled with mouse monoclonal antibody at 1:50 dilution. ZO-1 (Invitrogen, Carlsbad, CA, USA) was labeled with mouse monoclonal antibody at 1:100 dilution. ICAM-1 (Abcam, Cambridge, UK) was labeled with rabbit monoclonal antibody at 1:100 dilution. VCAM-1 (Abcam, Cambridge, UK) was labeled with rabbit monoclonal antibody at 1:250 dilution. MMP-9 (Abcam, Cambridge, UK) was labeled with rabbit monoclonal antibody at 1:250 dilution. After 1 h incubation at RT, cells were incubated with Alexa Fluor™ 488/594-conjugated secondary antibodies (1:1000 in PBS; Abcam, Cambridge, UK) and Phalloidin-iFluor^®^594 (1:1000 in PBS; Abcam, Cambridge, UK) for 1 h at RT in the dark. Finally, cells were treated with DAPI (Solarbio, Beijing, China) for 8 min at RT. Images were acquired using a Nikon fluorescence microscope or an Olympus confocal microscope.

### Vascular endothelial barrier permeability assay

After the HUVEC monolayer stabilized, apparent permeability (*P*_app_) of the vascular endothelial barrier was quantified using small molecules of fluorescein sodium and large molecules of 40 kDa and 70 kDa FITC-dextran (Sigma-Aldrich, St Louis, MO, USA). *P*_app_ was measured using phenol red-free medium containing 10 µg/mL fluorescein sodium, 12.5 µg/mL 40 kDa, and 12.5 µg/mL 70 kDa FITC-dextran. Medium was perfused through the upper channel at 5 μL/min flow rate. 50 μL samples were collected from the lower channel outlet at 15, 30, 45, and 60 min. Fluorescence emission was measured using a Horiba FluoroMax-4 spectrofluorometer with Orient KOJI Semi-Micro cuvettes. All steps were conducted at 25 ± 1°C. *P*_app_ was calculated using:


$$P_{\mathrm{app}}[\mathrm{cm}/s]=1/(A\times C_{0})\times (dQ/dt)$$

where *A* is the mass transfer area, *C*_0_ is the donor concentration in upper medium, and *dQ*/*dt* is the transmembrane transport rate. Barrier function and selective permeability were characterized by measuring *P*_app_ of different molecular weights across the vascular endothelial cell layer.

### Alkaline phosphatase and alizarin red staining

Following 7-day osteogenic differentiation of hBMSCs, early-stage mineralization was assessed via alkaline phosphatase (ALP) staining. Samples were fixed with 4% paraformaldehyde for 15 min at RT, then incubated with BCIP/NBT staining solution (BCIP: NBT: Alkaline Phosphatase Color Development Buffer = 1:2:300; Beyotime, Shanghai, China) in the dark at RT for 30 min. On Day 21 of osteogenic differentiation, late-stage mineralization was evaluated using Alizarin Red staining (Solarbio, Beijing, China). Samples were fixed with 4% paraformaldehyde for 15 min, stained with 0.2% Alizarin Red staining solution, and incubated in the dark at RT for 20 min. Images were acquired using a Nikon fluorescence microscope.

### Monocyte adhesion assay

Cell adhesion experiments were conducted using human monocytes (THP-1, ATCC, Manassas, VA, USA). Endothelial cells were labeled with Celltracker™ Green dye solution (Invitrogen, Carlsbad, CA, USA), while monocytes were stained with Celltracker™ Orange dye solution (Invitrogen, Carlsbad, CA, USA). Following 24 h pretreatment with or without LPS/TNF-α, monocytes (1 × 10^5^ cells/mL) were introduced into the chip and cultured at 37°C. After 2 h, the chip was removed to eliminate non-adherent cells and then imaged using a Nikon fluorescence microscope.

### Statistical analysis

Data were recorded using Excel (Microsoft) and analyzed statistically using GraphPad Prism^®^ 8. Differences between two groups were assessed by unpaired two-sided Student’s *t*-test. Multiple comparisons were performed using one-way analysis of variance (ANOVA) followed by Tukey’s *post hoc* test. Data are presented as mean ± SD, with statistical significance denoted as: *P < 0.05, **P < 0.01, ***P < 0.001.

## Results and discussion

### Reconstitution of bone-vessel interface-on-a-chip

#### On-chip formation and cell activity of a bone-vessel interface

Developing a periodontal chip that recapitulates physiological functions is critical for dissecting periodontitis pathogenesis and evaluating therapeutic strategies. In our previous work, we reported a periodontal soft tissue chip containing a gingival epithelial-capillary barrier that mimics periodontal anatomy and physiology while replicating soft tissue inflammation [39]. Alveolar bone, like other bone tissue, is a highly vascularized tissue: blood vessels deliver oxygen/nutrients, remove metabolites, and transport systemic signals (e.g. growth factors, neurotransmitters) to bone cells [46]. Therefore, we developed a bone-vessel interface chip, laying the foundation for the construction of a periodontal soft-hard tissue chip and investigating the circulatory effects of blood circulation on bone tissue in periodontitis.

The bone-vessel interface chip features a porous membrane dividing two chambers, serving as carriers or basement membranes for cell attachment and enabling bidirectional transport of drugs/metabolites through its microporous architecture [47]. HUVECs seeded in the upper chamber formed a functional vascular endothelial barrier, while hBMSCs cultured in the lower chamber underwent osteogenic differentiation. This design leverages HUVECs’ capacity for modeling endothelial inflammation and hBMSCs’ multipotency to generate COL-1 and osteocalcin (OCN)-secreting osteoblasts that initiate bone matrix synthesis (Figure 2D). Compared to traditional models, this microfluidic platform enables dynamic culture with precise environmental control and physiological/pathological microenvironment simulation, overcoming limitations of animal models (ethical constraints, interspecies variation) and static cultures (inadequate functional mimicry, inconsistent signaling gradients).

**Figure 3.**
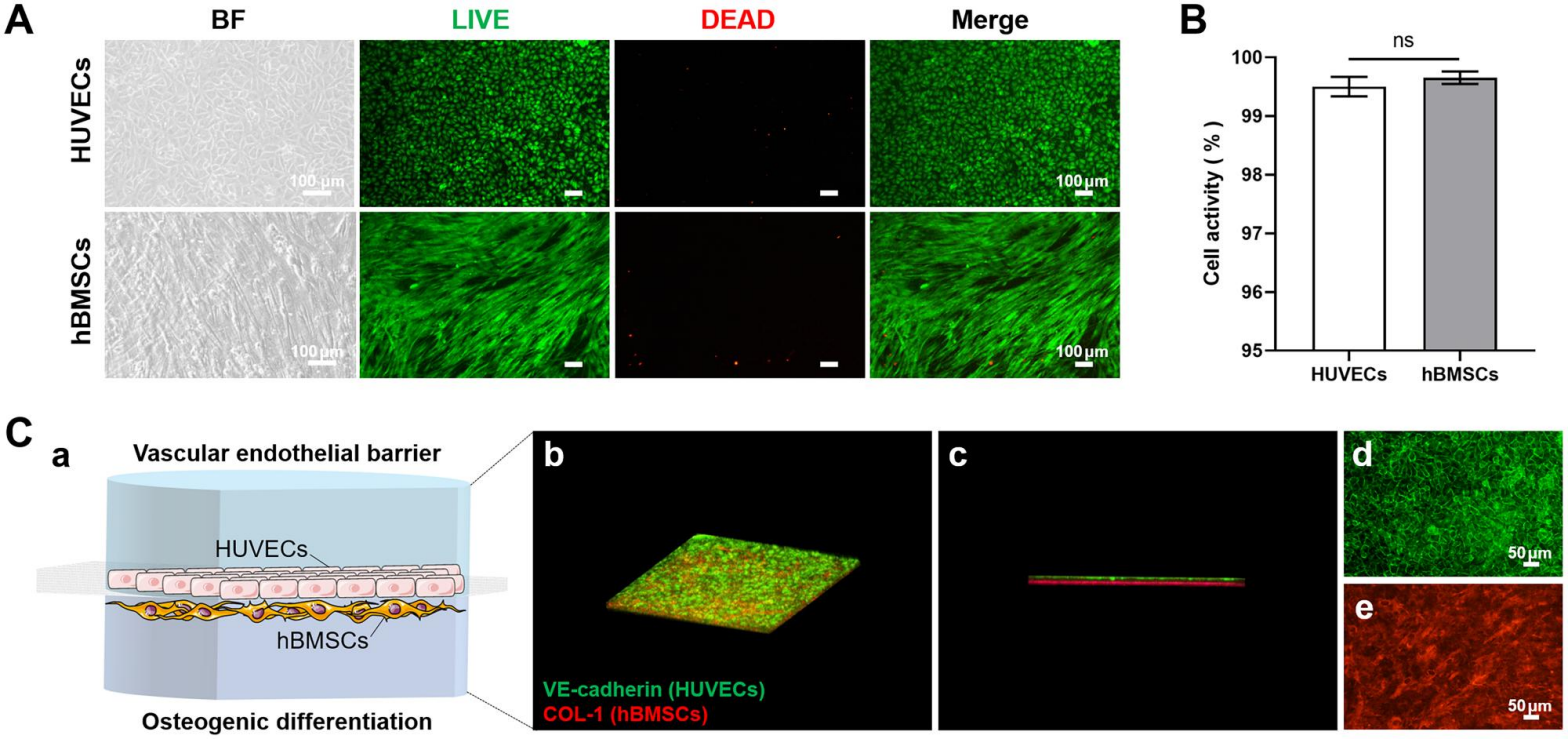
Bone-vessel interface-on-a-chip. (**A**) Bright-field and live/dead staining of hBMSCs and HUVECs cultured in the chip. Green: Living cells. Red: Dead cells. Scale bar = 100 μm. (**B**) Statistical analysis of cell viability. Data are means ± SD (*n* = 4). (**C**) 3D reconstruction of the schematic diagram (a), side (b and c), and top (d and e) views of a bone-capillary interface reconstruction. Green: VE-cadherin (HUVECs). Red: COL-1 (hBMSCs). Scale bar = 50 μm.

To validate chip biocompatibility and cellular suitability, we assessed cell viability via Live/Dead staining. Bright-field imaging revealed evenly distributed spindle-shaped hBMSCs and cobblestone-like HUVECs (Figure 3A). The cell viability was calculated based on the ratio of red to green areas in the image, and both hBMSCs and HUVECs exceeded 99% (Figure 3B). The cells cultured in the chip were observed under a low magnification microscope with complete morphology, most of which were living cells with green fluorescence. The fabricated PDMS chip platform was validated for multi-cell culture compatibility. As previously reported [48, 49], PDMS possesses key attributes—including biocompatibility, gas permeability, optical transparency, and thermal stability—that make it ideal for constructing biological models and microfluidic devices. Immunofluorescence staining of VE-cadherin and COL-1 confirmed the functionality of co-cultured cells. Confocal microscopy showed that VE-cadherin-expressing endothelial cells were located in the upper layer of the porous membrane, and COL-1-expressing mesenchymal stem cells were located in the lower layer of the porous membrane (Figure 3C). These results confirmed that hBMSCs and HUVECs in the chip have good cell viability. HUVECs can form a monolayer structure, and hBMSCs have the ability of osteogenic differentiation. Hence, the bone-vessel interface-on-a-chip simulates the structural relationship between blood vessels and bone tissue, which provides a research basis for further verifying the function of vascular endothelial barrier and osteogenic differentiation.

#### Function evaluation of vascular endothelial barrier in bone-vessel interface-on-a-chip

Vascular endothelial barrier originates from the physical connection at the cell–cell junctions, which critically regulate transport of fluids, solutes, and circulating cells [50]. Junction integrity can be characterized by analyzing connections between cytoskeletal elements and adhesion proteins. HUVEC-formed endothelial barrier exhibits selective permeability, serving as a key functional indicator. Material exchange assessment at vessel–tissue interfaces enables evaluation of organ-on-chip physiological relevance. VE-cadherin, as an endothelial-specific cadherin, is essential for junction formation and regulation. Through F-actin interaction, it governs vascular permeability, structural integrity, and stability [51]. Furthermore, the mechanical forces exerted by blood flow on vascular cells are critical signals for maintaining cellular function and homeostasis. This mechanical stimulation can influence vascular functional status and gene expression patterns by altering the morphology and proliferative activity of endothelial cells [52, 53]. Among these, the physiological shear stress associated with continuous unidirectional laminar flow can significantly enhance cell–cell junctions and cell–matrix adhesion, thereby reducing vascular permeability [54]. Using microfluidic technology, the structural and functional characteristics of the vascular endothelial barrier can be accurately recapitulated *in vitro* through perfusion culture [55, 56]. Based on this, the present study systematically evaluates the structural integrity and functional properties of the vascular endothelial barrier in the bone-vessel interface-on-a-chip.

**Figure 4.**
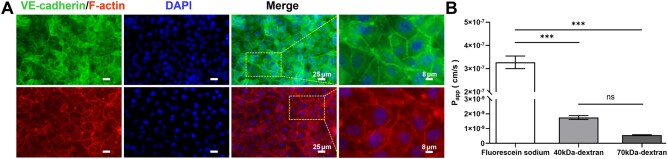
Characterization of HUVECs in bone-vessel interface-on-a-chip. (**A**) Representative fluorescent images of VE-cadherin (green), cytoskeleton (red; stained with F-actin), and DAPI (blue) of HUVECs. (**B**) Apparent permeability (*P*_app_) of fluorescein sodium, 40 kDa, and 70 kDa FITC−dextran through the HUVEC layer. Data are means ± SD (*n* = 4).

In this study, HUVECs cultured on the porous membrane of the chip were stained with VE-cadherin and F-actin. Fluorescence images were taken and observed at 40×. As shown in Figure 4A, endothelial cells were evenly distributed on the porous membrane with normal morphology. High VE-cadherin expression at intercellular junctions confirmed stable barrier formation with functional characteristics. Molecular transport varied by size: small-molecule fluorescein sodium (376.27 Da) freely diffused across the barrier, while large-molecule FITC-dextrans (40 and 70 kDa) exhibited restricted passage. For the vascular endothelial barrier formed by HUVECs, the 70 kDa FITC-dextran has the lowest *P*_app_, followed by 40 kDa, and both *P*_app_ were smaller than 10^−7^. Meanwhile, the *P*_app_ of fluorescein sodium has the highest Papp, which was larger than 10^−7^. The difference in *P*_app_ among the three was consistent with the difference in molecular weight (Figure 4B), demonstrating that the HUVEC monolayer recapitulates the *in vivo* vascular barrier’s ability to permit small-solute passage while restricting macromolecules. Collectively, these results confirm that the chip’s endothelial barrier exhibits molecular weight-dependent selective permeability, analogous to native capillary function.

#### Function evaluation of osteo-differentiated hBMSCs in bone-vessel interface-on-a-chip

**Figure 5.**
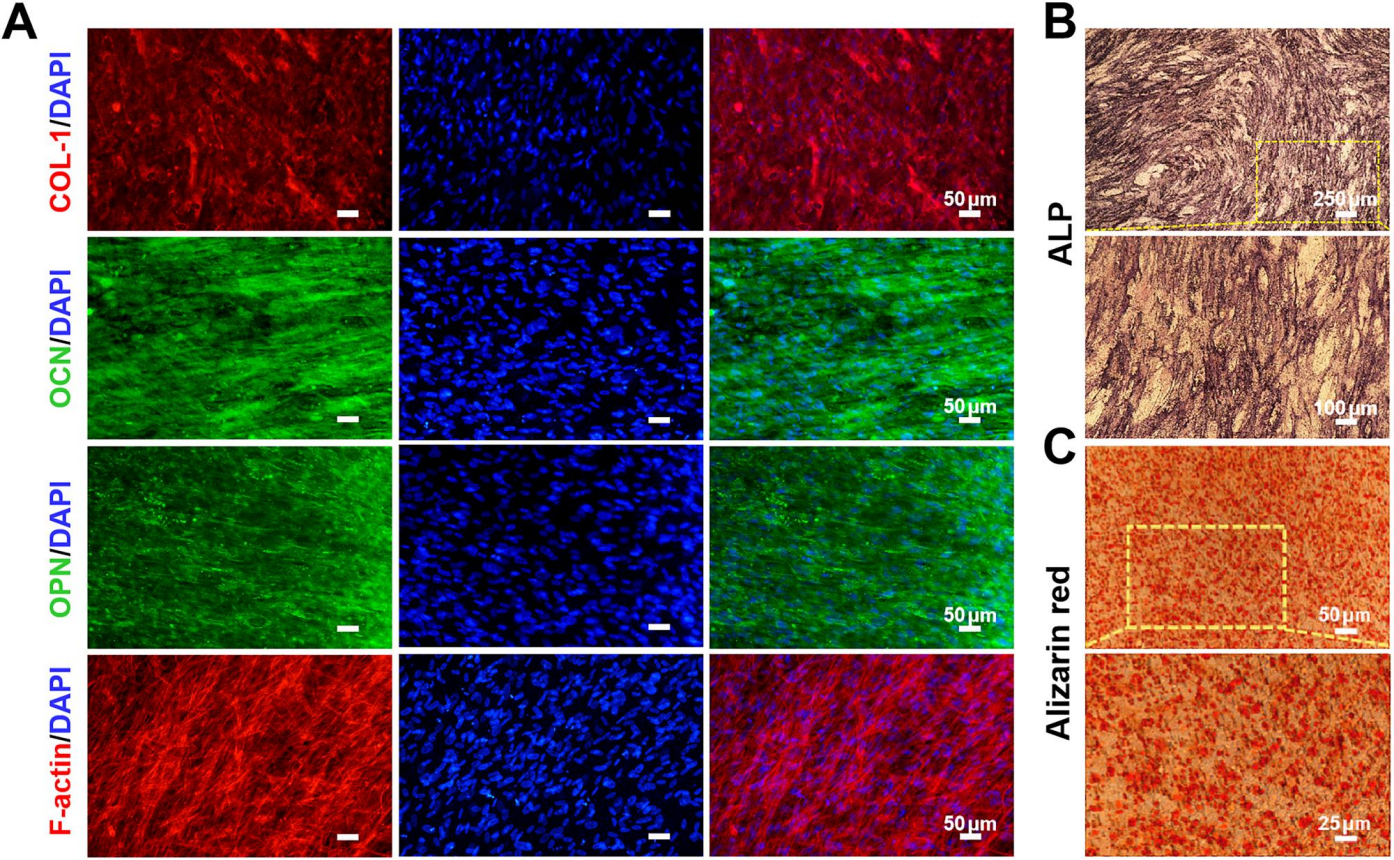
Characterization of osteo-differentiated hBMSCs in bone-vessel interface-on-a-chip. (**A**) Immunofluorescent images of COL-1 (red), OCN (green), OPN (green), F-actin (red), and DAPI (blue) of hBMSCs after induction of osteogenic differentiation for 14–21 days. Scale bar = 50 μm. (**B**) ALP staining images after 7 days of osteogenic differentiation. (**C**) Alizarin red staining images after 21 days of osteogenic differentiation.

Bone tissue consists of diverse cell types embedded within an ECM, which is primarily divided into organic and inorganic components. The organic components mainly consist of COL-1 and osteocalcin (OCN), while the inorganic components mainly consist of calcium-phosphate-based hydroxyapatite. Among these, the middle-to-late markers of osteoblast differentiation and bone formation, including COL-1, OCN, and osteopontin (OPN), serve as critical indicators of osteogenic functional maturation [57]. These organic matrix components play pivotal roles in bone development. In this study, hBMSCs were seeded on the lower layer of the porous membrane and subjected to osteogenic differentiation for 14–21 days, followed by immunofluorescence staining for bone-related proteins. As shown in Figure 5A, osteogenically induced hBMSCs synthesized and secreted COL-1, OCN, and OPN. F-actin and DAPI staining showed robust actin polymerization and prominent stress fiber formation in hBMSCs post-osteogenic differentiation.

ALP, an early sign of osteoblast differentiation and functional maturation, plays a role in promoting mineralization. ALP staining was performed on Day 7 to evaluate early osteogenic differentiation and was observed to be positive (Figure 5B). During mineralization, ALP facilitates calcium ion deposition on collagen fibers. Alizarin red can chelate with calcium ions to stain intracellular calcium deposits and the mineralized nodules, resulting in red complexes. On Day 21, alizarin red staining was used to evaluate the late osteogenic differentiation. Results are shown in Figure 5C, calcification was formed in the cells and mineralized nodules were formed. The osteo-differentiated hBMSCs in the chip exhibit characteristics of mature osteoblasts, including bone-related protein secretion and mineralized matrix formation.

These findings establish that the bone-vessel interface-on-a-chip recapitulates key native tissue features: endothelial cells form a monolayer barrier with selective permeability, while osteo-differentiated hBMSCs can secrete bone-related proteins and form mineralization. Mature osteoblasts were observed in the chip, thus providing the potential to further study the pathological mechanism of periodontitis by the proposed platform.

### LPS and TNF-α induced periodontal inflammation in bone-vessel interface-on-a-chip

#### Vascular endothelial barrier dysfunction

**Figure 6.**
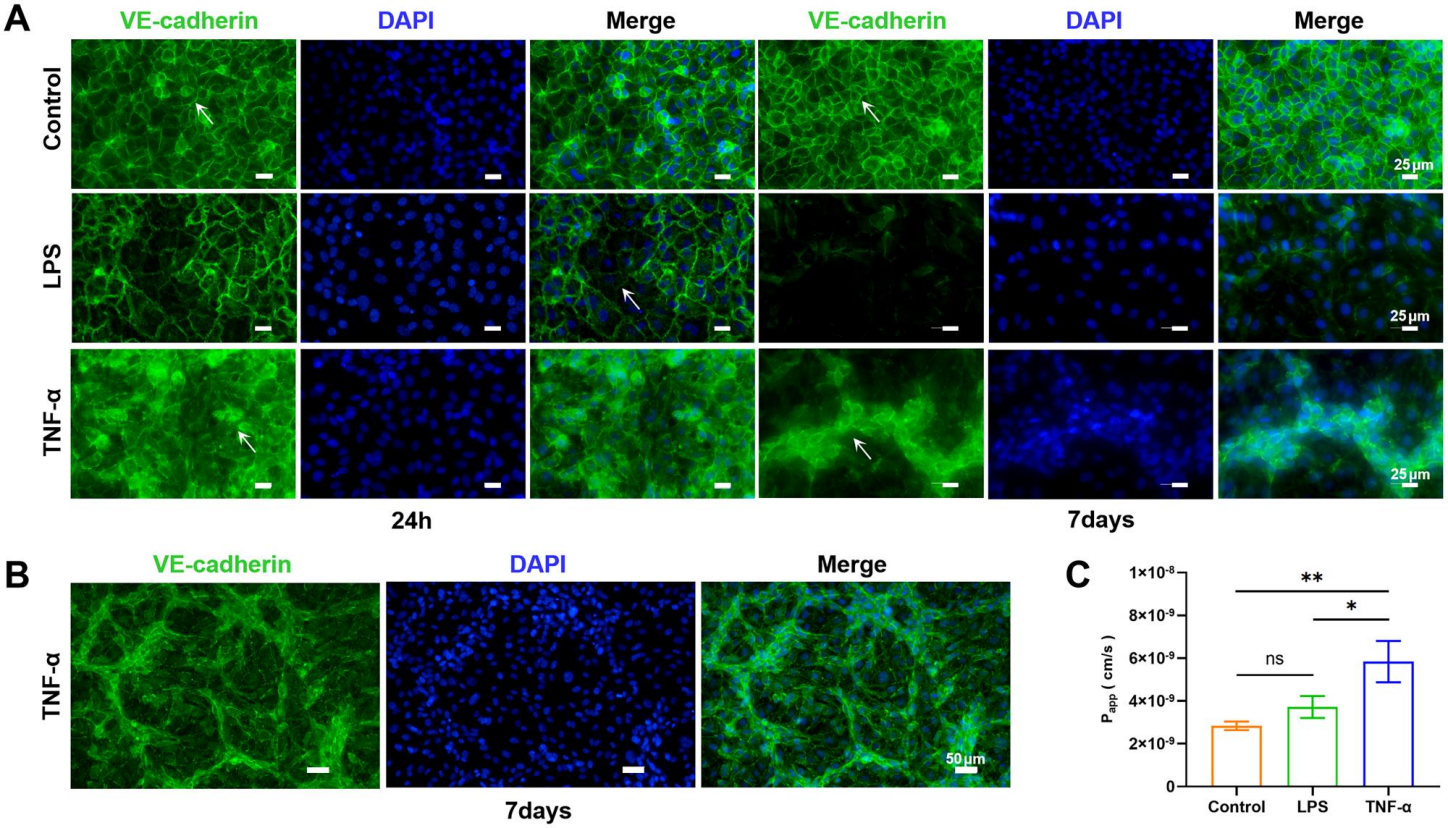
Vascular endothelial barrier dysfunction. (**A**) Fluorescent images showed the expression of VE-cadherin in endothelial cells after LPS or TNF-α treatment for 24 h or 7 days on-chip. Arrows (white) indicate that in the LPS and TNF-α groups, the vascular barrier structure was disrupted, while the control group maintained a normal barrier structure. Scale bar = 25 μm. (**B**) Abnormal vascular proliferation was observed after 7 days of TNF-α treatment. Scale bar = 50 μm. (**C**) Apparent permeability (*P*_app_) using 70 kDa FITC−dextran for control, LPS, or TNF-α. Data are means ± SD (*n* = 3).

Endothelial cells directly participate in defense against inflammation and infection, serving as the first line of defense. Periodontitis pathogenic bacteria/metabolites and pro-inflammatory factors enter the blood circulation, which leads to endothelial cell dysfunction, disrupts the tight junctions between endothelial cells, causes increased vascular barrier permeability, and results in fluid and protein leakage through the vascular wall [50, 58]. Meanwhile, reduced endothelial barrier function is central to the long-term inflammatory response. Adhesion junctions are protein complexes that form cell–cell interactions. VE-cadherin is specifically expressed in endothelial cells and is considered to be the central part of adhesion junctions structurally and functionally [50]. To characterize the structural and functional effects of the *P. gingivalis* metabolite LPS and pro-inflammatory factor TNF-α on the vascular endothelial barrier, we first evaluated VE-cadherin expression. Compared with the control group, after 24 h of LPS or TNF-α treatment, the endothelial cell arrangement was changed, VE-cadherin expression disappeared in some regions, and intercellular junctions were disrupted (Figure 6A). Similar results were observed in fluorescence images of the tight junction protein ZO-1, with loss of tight junctions between endothelial cells in the LPS and TNF-α groups (Supplementary Figure S1). After 7 days, VE-cadherin expression was further decreased, and endothelial cells were remodeled morphologically and became larger, predicting a possible further increase in vascular endothelial barrier permeability (Figure 6A). In contrast, endothelial cells in the control group remained in good morphology and arranged in a monolayer, and the vascular endothelial barrier structure was intact. Interestingly, the TNF-α group exhibited abnormal vascular proliferation with VE-cadherin overexpression on these structures (Figure 6B; Supplementary Figure S2). Inflammatory stimuli can disrupt the junctions between endothelial cells, leading to abnormally increased vascular permeability and subsequent excessive extravasation of macromolecular substances [59]. This process is not only a direct sign of impaired endothelial cell integrity but also a key pathological feature of amplified inflammatory responses. Permeability assays showed that the permeability of 70 kDa dextran increased after 7 days of LPS and TNF-α treatment, with a more significant increase in the TNF-α group compared to the control group, indicating disruption of the vascular endothelial barrier integrity (Figure 6C). Abnormal changes in vascular endothelial barrier function hold significant pathological significance in disease progression: on one hand, the disruption of barrier integrity increases the risk of pathogenic bacteria (such as periodontal pathogens) entering the bloodstream, which may trigger distant tissue infections or systemic inflammatory responses; on the other hand, overly permeable vascular endothelium also promotes the abnormal recruitment of white blood cells, exacerbating inflammatory damage to local tissues [60]. Therefore, regulating vascular permeability (e.g. repairing inter-endothelial junctions, inhibiting overactivated inflammatory signaling pathways) can reduce macromolecular extravasation and abnormal infiltration of white blood cells to alleviate inflammatory symptoms, providing a new potential target and strategy for the prevention and treatment of inflammatory diseases.

VE-cadherin critically maintains endothelial contact integrity through homotypic associations and preserves barrier function [61]. Reduced VE-cadherin expression following *P. gingivalis* metabolite LPS and pro-inflammatory factor TNF-α exposure indicate compromised cell–cell contact integrity. Observed abnormal vascular proliferation in the TNF-α group may stem from endothelial cell proliferation and migration [62]. VE-cadherin also regulates angiogenesis [63, 64], explaining its overexpression at abnormal vascular sites. These findings align with previously published theories. Literature has proposed that, unlike acute inflammation causing transient junctional disruption, chronic inflammation (such as periodontitis) induces vascular remodeling featuring enlargement, proliferation, sustained gap formation and leakage, and these changes do not spontaneously resolve [65]. Thus, vascular barrier structural disruption can serve as a pathophysiological marker in chronic inflammation, and enhancing vascular barrier function may represent a novel therapeutic strategy to counteract potential host adaptive adverse responses to inflammation.

#### Regulation of transendothelial migration by endothelial cells

**Figure 7.**
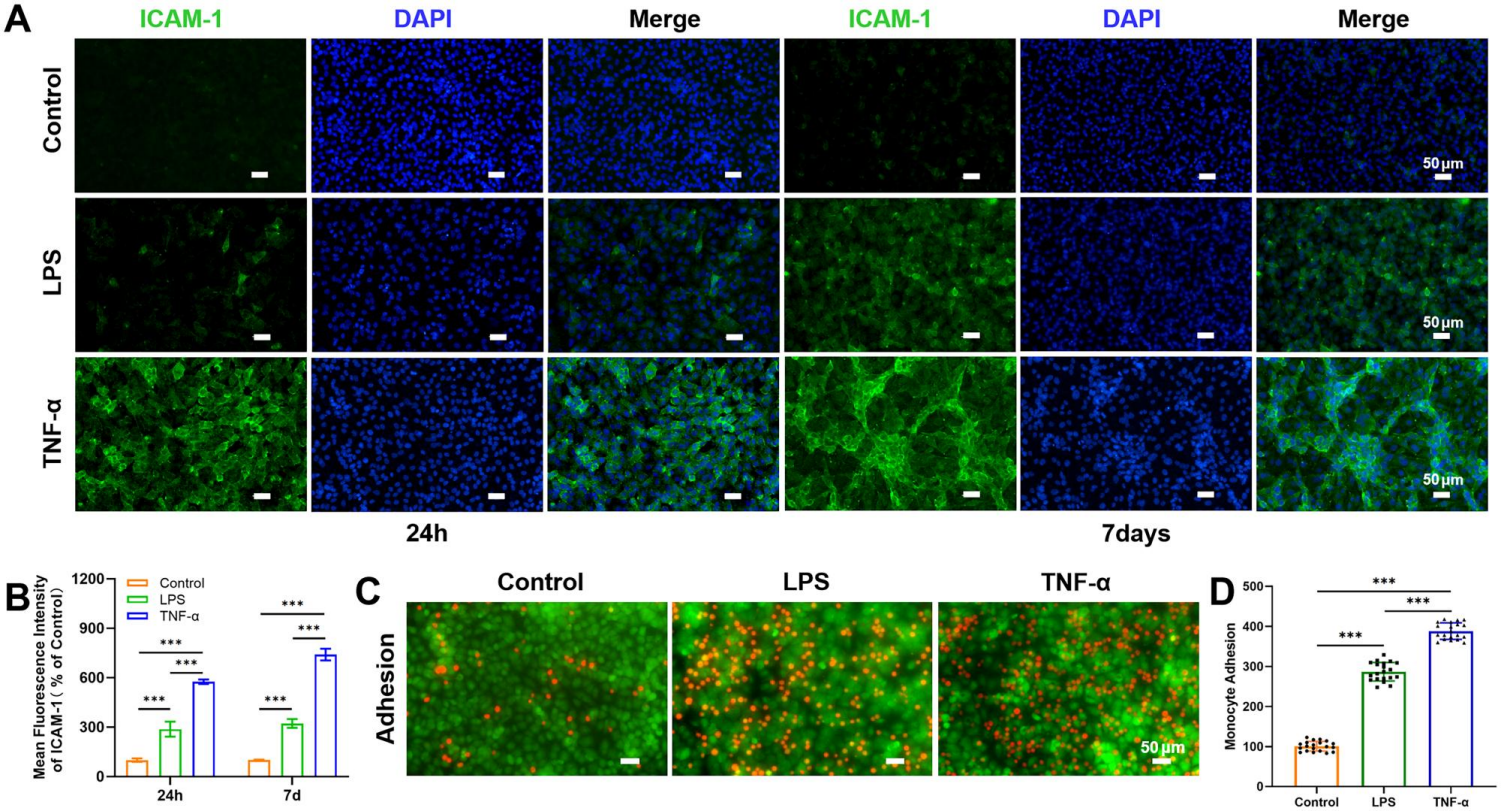
Regulation of transendothelial migration by endothelial cells. (**A**) Fluorescent images showed the expression of ICAM-1 in endothelial cells after LPS or TNF-α treatment for 24 h or 7 days on-chip. Scale bar = 50 μm. (**B**) Quantitative fluorescence analysis of ICAM-1 in each group. Data are means ± SD (*n* = 3). (**C**) Image showing increased monocyte (Celltracker™ orange) adhesion on endothelial cell (Celltracker™ green) surface after LPS or TNF-α treatment on-chip. Scale bar = 50 μm. (**D**) Quantitative analysis of the number of monocyte adhesions. Data are means ± SD (*n* = 4).

Adhesion molecules are considered prerequisites for inflammatory leukocyte TEM and promote leukocyte adhesion [50]. Under inflammatory conditions, endothelial cells upregulate adhesion molecule expression [8]. Therefore, the expression of adhesion factor ICAM-1 was first assessed. After 24 h and 7 days of LPS or TNF-α treatment, compared with the control group, ICAM-1 expression was significantly increased in the TNF-α group, followed by the LPS group (Figure 7A and B). Notably, after 7 days of TNF-α exposure, ICAM-1 was highly expressed in areas of abnormal vascular proliferation. Furthermore, the effects of LPS and TNF-α on the adhesion of monocytes in the chip were investigated. Monocytes were labeled with red fluorescence and endothelial cells with green fluorescence, and adherent monocytes were counted. As shown in Figure 7C, both LPS and TNF-α significantly increased monocyte adhesion, with the TNF-α group exhibiting the highest number of adherent monocytes (3.9-fold increase over control), followed by the LPS group (2.8-fold increase) (Figure 7D). These data suggest a positive correlation between ICAM-1 expression levels and monocyte adhesion: the TNF-α group displayed the strongest ICAM-1 upregulation and the highest adhesion capacity. This confirms that LPS and TNF-α promote monocyte adhesion by activating endothelial cells and increasing ICAM-1 expression.

Growing evidence demonstrates that periodontitis can initiate and exacerbate systemic diseases via the bloodstream, including cardiovascular diseases such as atherosclerosis [66, 67]. In our study, LPS/TNF-α were employed to simulate vascular endothelial barrier dysfunction during periodontitis pathogenesis, characterized by disruption of interendothelial junctions, increased permeability, and enhanced monocyte adhesion. These findings are consistent with pathological manifestations of early endothelial injury in atherosclerosis [68]. Meanwhile, inflammation plays a crucial role in atherosclerosis pathogenesis and progression. Most therapeutic strategies for vascular endothelial barrier dysfunction primarily target known risk factors rather than specifically addressing endothelium-based mechanisms [69]. Studies on atherosclerosis indicate that endothelial cells undergo phenotypic transformation during disease progression. This transformation occurs through endothelial-to-mesenchymal transition (EndMT), impairing endothelial function and inducing vascular remodeling, potentially serving as a driving factor for inflammatory progression [70, 71]. Evidence has shown that statins can improve endothelial function by epigenetically inhibiting EndMT [72]. This may offer new insights for investigating related pathological mechanisms and developing therapeutic strategies.

#### Inflammatory response of bone-vessel interface triggered by TNF-α

**Figure 8.**
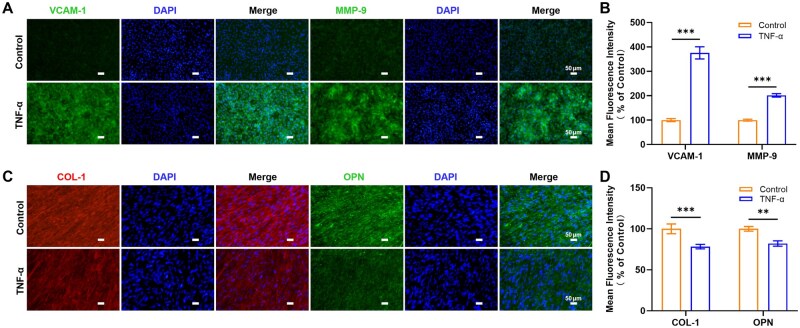
Further studies on the inflammatory response caused by TNF-α. (**A**) Fluorescent images showed the expression of VCAM-1 and MMP-9 in endothelial cells after TNF-α treatment for 24 h. Scale bar = 50 μm. (**B**) Quantitative fluorescence analysis of VCAM-1 and MMP-9. Data are means ± SD (*n* = 3). (**C**) Fluorescent images showed the expression of COL-1 and OPN in osteo-differentiated hBMSCs after TNF-α treatment for 14 days. Scale bar = 50 μm. (**D**) Quantitative fluorescence analysis of COL-1 and OPN. Data are means ± SD (*n* = 3).

To further investigate the role of TNF-α in regulating the vascular endothelial barrier through this platform, we analyzed the expression of VCAM-1 and MMP-9. After 24 h of TNF-α treatment, the expressions of VCAM-1 and MMP-9 in the TNF-α group were significantly increased compared with the control group (Figure 8A and B). Similar to ICAM-1, upregulation of VCAM-1 (as an adhesion molecule) enhances inflammatory leukocyte TEM. MMPs, particularly MMP-9 and MMP-13, are secreted by osteoclasts and endothelial cells to promote cell proliferation, migration, and differentiation. They play roles in vascular remodeling, cell apoptosis, and tissue repair [61]. This may explain the abnormal vascular proliferation observed in the TNF-α group.

TNF-α suppresses osteoblastogenesis during the differentiation of pre-osteoblasts [73]. Its mechanism of action involves the synergistic effect of multiple signaling pathways. TNF-α can either activate the NF-κB pathway to downregulate EphB4 signaling or upregulate SOX5 and mediate the KLF4 signaling pathway to inhibit the expression of RUNX2, thereby reducing ALP activity and the formation of mineralized nodules [74–77]. At the same time, it induces osteoblast apoptosis, ultimately leading to bone loss [78]. Control groups showed secretion of bone-related proteins COL-1 and OPN, while TNF-α treatment decreased their expression (Figure 8C and D). During the pathogenesis of periodontitis, host immune cells are activated to produce pro-inflammatory factors like TNF-α. Sustained TNF-α exposure disrupts normal bone remodeling, inhibiting osteoblast activity and bone matrix protein production. Concomitantly, inhibition of osteoblast bone formation and osteocyte survival, coupled with stimulation of osteoclast-mediated bone resorption, leads to reduced bone volume. These results demonstrate that the bone-vessel interface-on-a-chip can recapitulate the specific functions of the vascular barrier and bone *in vitro*, and respond to periodontal pathogenic bacterial metabolites and pro-inflammatory factors. It successfully simulates vascular barrier dysfunction, subsequent leukocyte adhesion, and downstream bone tissue impacts in the periodontitis microenvironment.

Periodontal tissues (including the gingiva, alveolar bone, and periodontal ligament) are highly vascularized. Svanberg *et al*. [34] reported that periodontal ligament cells (PDLCs) maintained a round morphology under static monoculture conditions without HUVECs. This suggests that the morphological transition of PDLCs to elongated sprout-like shapes may be associated with factors such as co-culture with HUVECs, growth factors in endothelial culture media, and shear stress. Thus, it is evident that incorporating endothelial cells into model construction is necessary. Furthermore, as the oral cavity is a system that directly interacts with the outside world, some models incorporate the protective effects of gingival crevicular fluid (GCF) flow and saliva buffering on the host. Simulated GCF flow attenuates inflammatory mediator secretion, potentially by reducing sustained pathogen-associated molecular pattern exposure that triggers excessive inflammatory responses [79]. Simultaneously, simulated salivary buffering yields denser gingival epithelial barrier architecture and significantly enhanced barrier function [80].

Previous studies modeled periodontitis using periodontal pathogens, LPS, Toll-like receptor 2 (TLR2) and TLR4 agonists, and pro-inflammatory factors to construct models of the gingiva, periodontal ligament, and connective tissue for investigating host–microbe interactions in the periodontium [33, 34, 79–81]. These studies, through inflammation disease simulation, observed increased secretion of pro-inflammatory factors and chemokines, as well as upregulated expression of adhesion proteins. Unlike these previous studies, our study focuses more on observing structural and functional alterations of the vascular endothelial barrier in inflammatory environments. Although LPS and TNF-α induce inflammation with controllability and repeatability, recapitulating typical features of periodontal inflammation, they still represent simplified models that cannot fully mimic the complex pathological manifestations of direct pathogen action *in vivo*. Therefore, future model construction should not only integrate multiple periodontal tissue components but also comprehensively consider multidimensional factors such as immune responses, inflammatory microenvironment simulation methods, and fluid dynamics to establish more comprehensive disease models.

Based on the aforementioned understanding of the complexity involved in constructing periodontal tissue models and the need for multi-dimensional integration, current research has shown a tendency toward adopting microfluidic platforms in terms of technical platform selection and model design. However, existing models are often limited to observing responses of a single tissue or lack integrated vascular components, which greatly restricts the simulation of systemic pathological cascades. Our bone-vessel interface-on-a-chip has made progress in this field: it recapitulates vascular barrier dysfunction and reduced bone tissue-related proteins under inflammatory conditions. This model provides a novel platform for the prevention and treatment of periodontitis, which can be used for: (i) drug screening by evaluating the ability of therapeutic drugs to reverse endothelial barrier protection or osteogenesis promotion; (ii) investigating new interventions (e.g. therapeutic drugs targeting endothelial cells) to prevent periodontitis-associated systemic complications by improving vascular barrier function. This bone-vessel interface-on-a-chip can effectively study the effects of periodontitis metabolites and pro-inflammatory factors on the vascular barrier and bone tissue through the controlled integration of biochemical and biophysical cues, thus providing a valuable tool for researching targeted therapies for endothelial cell-related inflammatory diseases, including periodontitis and its associated systemic diseases.

## Conclusion

In conclusion, we developed a biomimetic bone-vessel interface-on-a-chip using hBMSCs and HUVECs. This platform successfully recapitulated a selectively permeable vascular endothelial barrier and osteoblasts capable of secreting bone-related proteins to form mineralized matrix. Upon exposure to *P. gingivalis* metabolite LPS and pro-inflammatory factor TNF-α, the model exhibited vascular endothelial junction disruption, upregulated adhesion protein expression, enhanced monocyte adhesion, impaired barrier function, and reduced bone-related protein expression. These findings demonstrate that the chip can effectively simulate functional, structural, and pathological alterations in the vascular barrier and bone under physiological and inflammatory conditions. Like advanced microfluidic systems, this model incorporates organ-specific functional recapitulation, dynamic perfusion culture, and physiological/pathological microenvironment simulation. Thus, the bone-vessel interface-on-a-chip may serve as a versatile platform for investigating complex biological phenomena under controlled biochemical/biophysical cues, offering significant potential as an alternative to traditional preclinical models. It provides a robust foundation for future studies, including integration of periodontal soft-hard tissue chip, the study of periodontitis pathogenic bacteria/metabolites and pro-inflammatory factors that directly and indirectly induce or aggravate other systemic diseases through the blood circulatory system, as well as the treatment of inflammatory diseases by targeting endothelial cells.

## Supplementary Material

rbaf111_Supplementary_Data
