# Supplementary material for: Biomimetic bone-vessel interface-on-a-chip for simulating periodontal physiological and pathological microenvironment
Source: Regen Biomater. 2025 Oct 28;12:rbaf111. doi: 10.1093/rb/rbaf111 (PMC12714388; doi:10.1093/rb/rbaf111)
Supplement: rbaf111_Supplementary_Data [file rbaf111_supplementary_data.zip › Supplementary data.docx]

**Supplementary data**


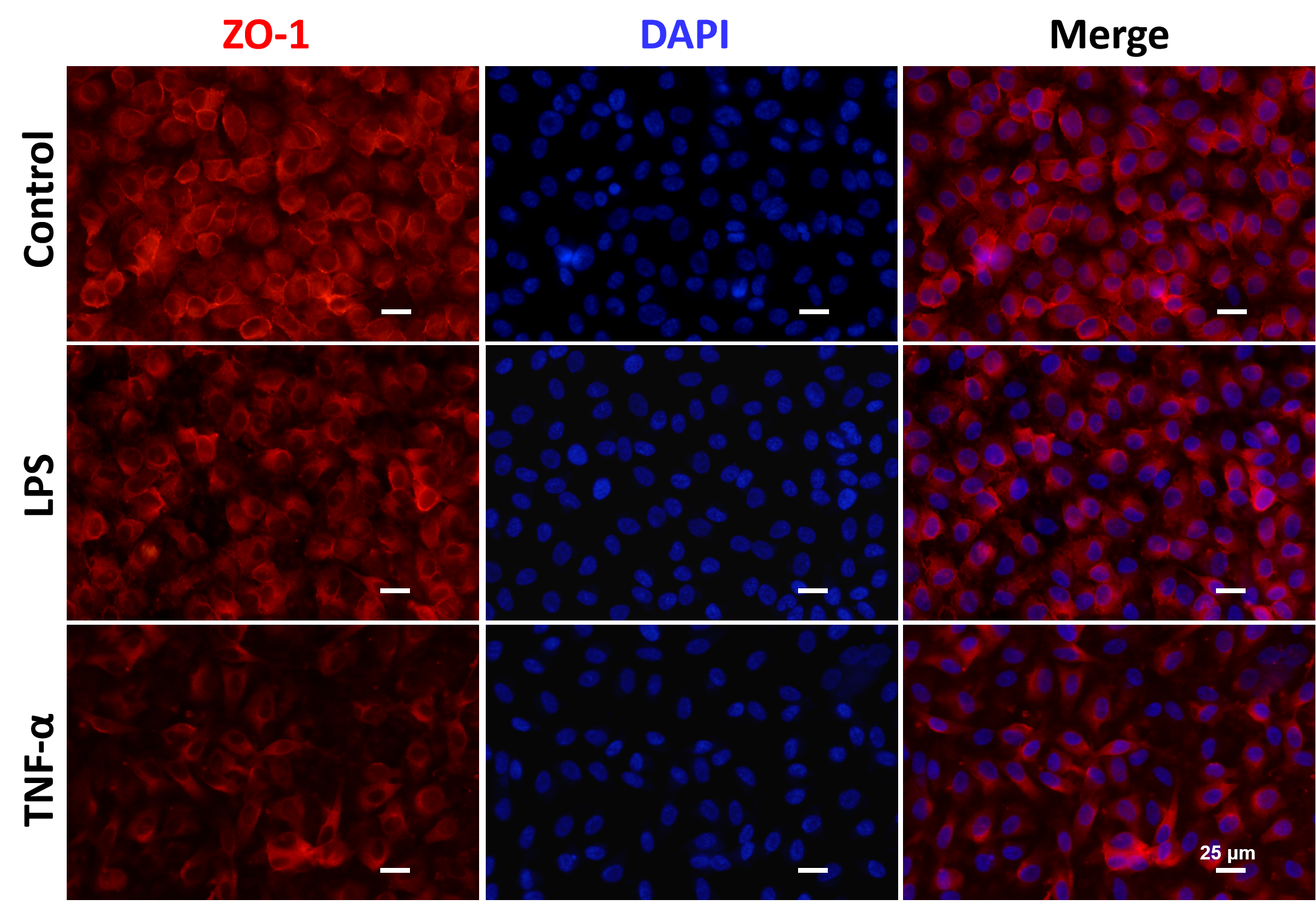


Supplementary Figure 1. Fluorescence images of the tight junction protein ZO-1 in endothelial cells after LPS or TNF-α treatment for 24 h. Scale bar = 50 μm.


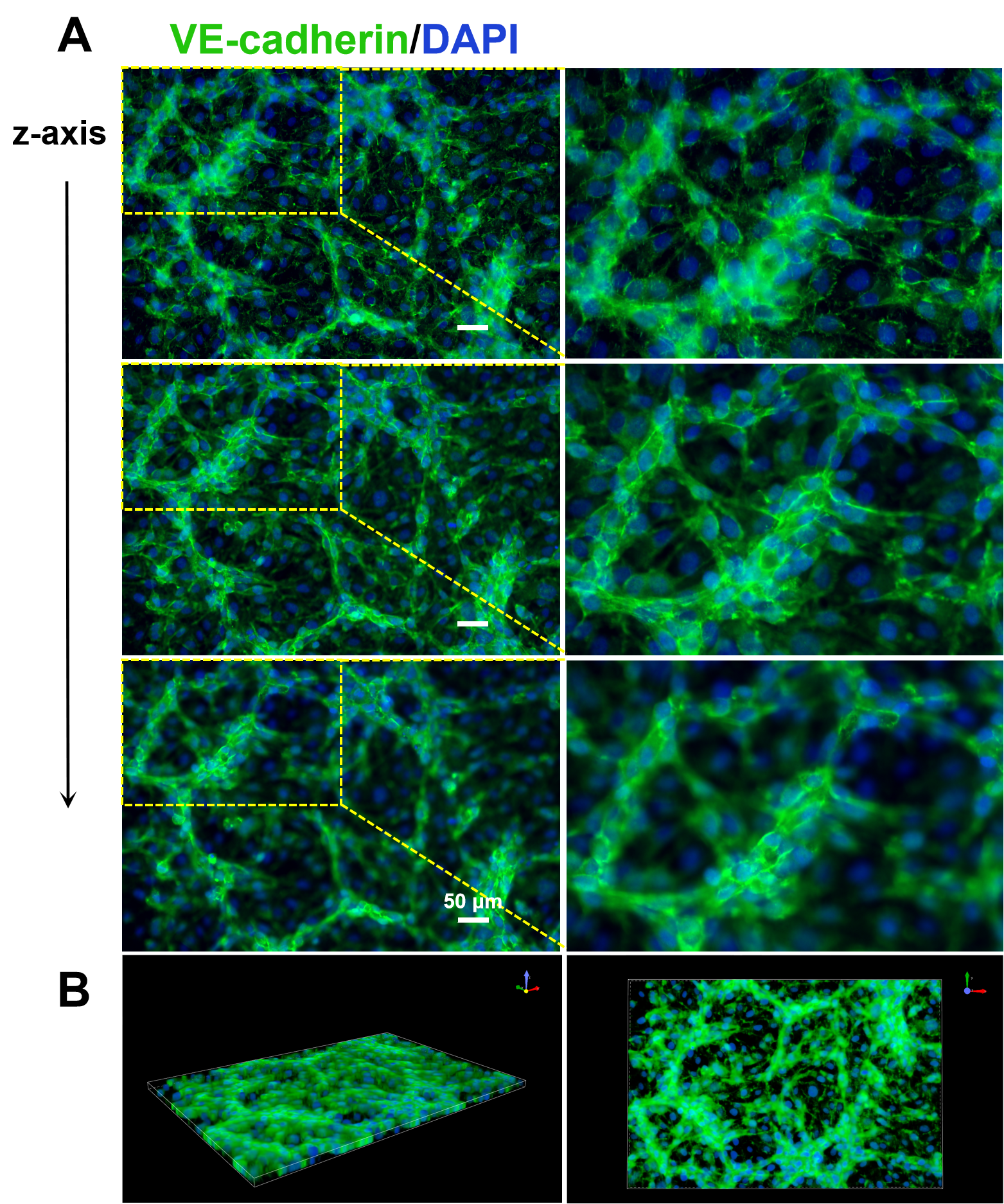


Supplementary Figure 2. Abnormal angiogenesis was observed after 7 days of TNF-α treatment. A. As the Z-axis changes, it is observed that the expression level of VE-cadherin is lower in the vascular endothelial barrier, while it is higher within the abnormal vascular proliferation areas. The expression of VE-cadherin in the abnormal vessel proliferation area shows spatial variation along the Z-axis, indicating that the abnormal vessel proliferation site and the constructed vascular barrier exist in different lesion planes. Scale bar = 50 μm. The processed image is shown in Figure 6B. B. Three-dimensional reconstruction image after VE-cadherin staining.
