# Supplementary figures and images for: Biomimetic bone-vessel interface-on-a-chip for simulating periodontal physiological and pathological microenvironment
Source: Regen Biomater. 2025 Oct 28;12:rbaf111. doi: 10.1093/rb/rbaf111 (PMC12714388; doi:10.1093/rb/rbaf111)

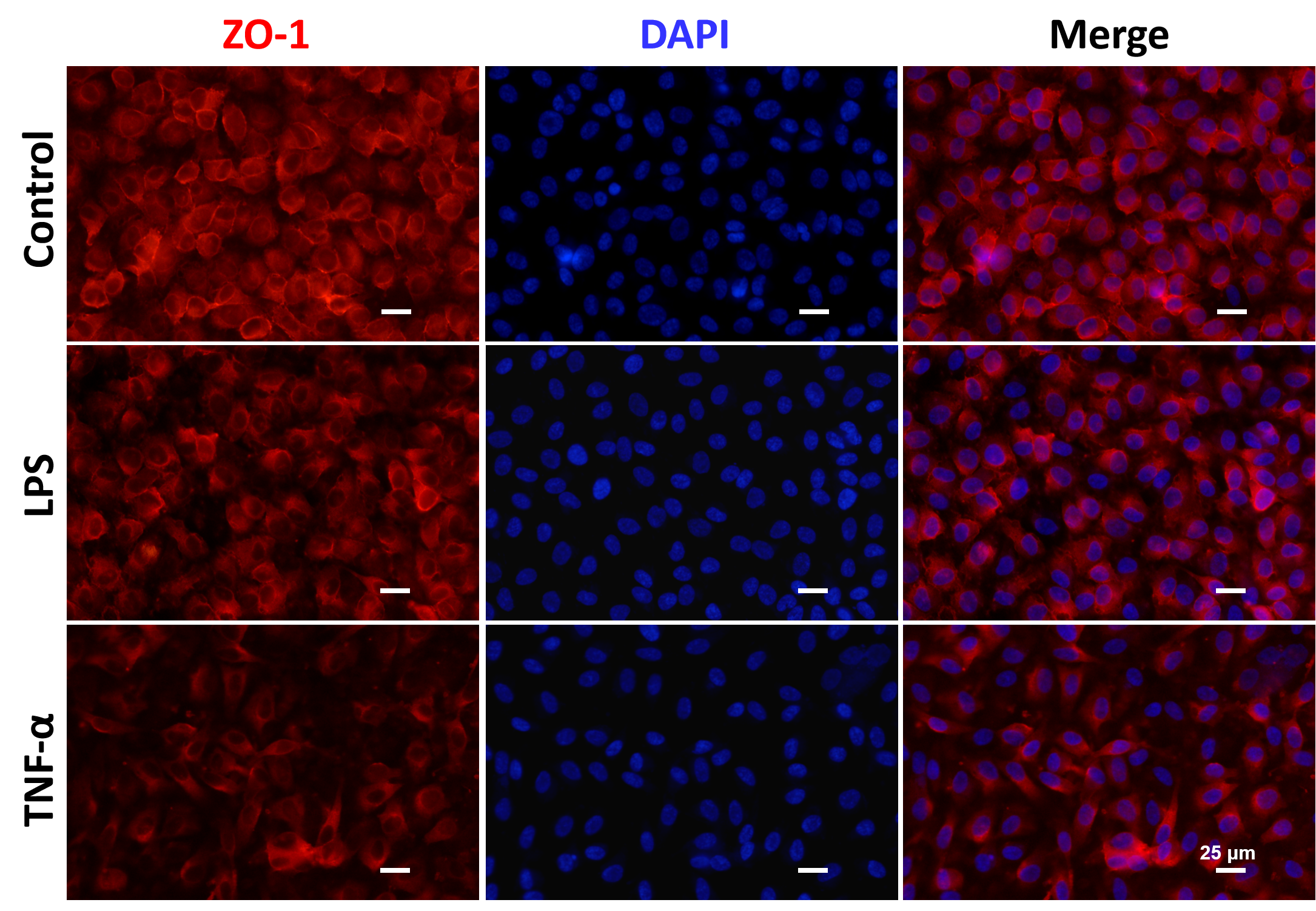

Supplement: rbaf111_Supplementary_Data [file rbaf111_supplementary_data.zip › Supplementary Figure 1.png]

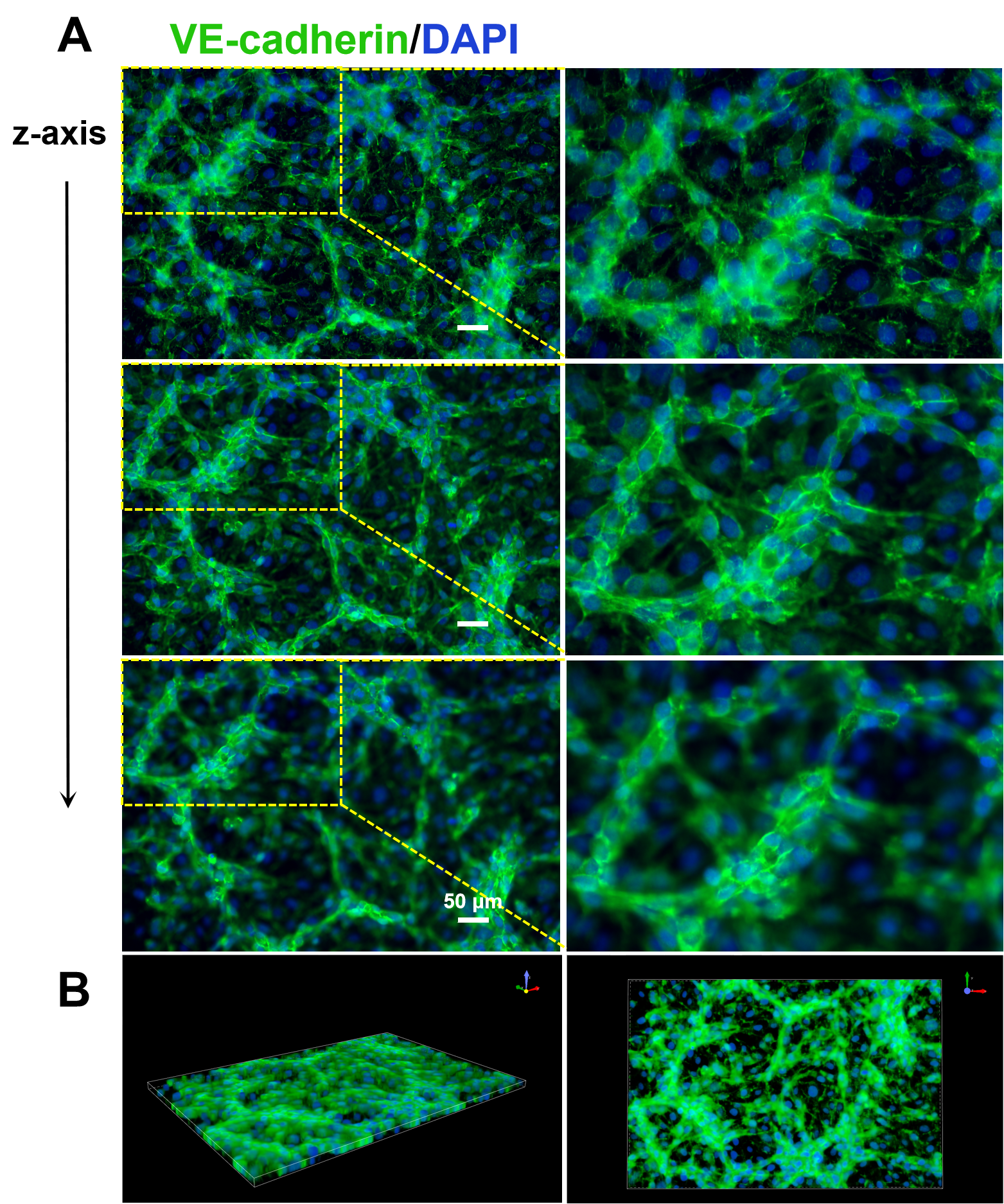

Supplement: rbaf111_Supplementary_Data [file rbaf111_supplementary_data.zip › Supplementary Figure 2.png]
